# FMCW Laser Fuze Structure with Multi-Channel Beam Based on 3D Particle Collision Scattering Model under Smoke Interference

**DOI:** 10.3390/s24165395

**Published:** 2024-08-21

**Authors:** Zhe Guo, Bing Yang, Kaiwei Wu, Yanbin Liang, Shijun Hao, Zhonghua Huang

**Affiliations:** School of Mechatronical Engineering, Beijing Institute of Technology, Beijing 100081, China; 3120185119@bit.edu.cn (Z.G.); 3120215164@bit.edu.cn (B.Y.); 3120205163@bit.edu.cn (K.W.); 3120195127@bit.edu.cn (Y.L.); 3120215165@bit.edu.cn (S.H.)

**Keywords:** FMCW laser fuze, smoke particle interference, 3D particle collision scattering, particle feature co-simulation, multiple channel laser emission, Unity3D

## Abstract

In the environment of smoke and suspended particles, the accurate detection of targets is one of the difficulties for frequency-modulated continuous-wave (FMCW) laser fuzes to work properly in harsh conditions. To weaken and eliminate the significant influence caused by the interaction of different systems in the photon transmission process and the smoke particle environment, it is necessary to increase the amplitude of the target echo signal to improve the signal-to-noise ratio (SNR), which contributes to enhancing the detection performance of the laser fuze for the ground target in the smoke. Under these conditions, the particle transmission of photons in the smoke environment is studied from the perspective of three-dimentional (3D) collisions between photons and smoke particles, and the modeling and Unity3D simulation of FMCW laser echo signal based on 3D particle collision is conducted. On this basis, a laser fuze structure based on multiple channel beam emission is designed for the combined effect of particle features from different systems and its impact on the target characteristics is researched. Simulation results show that the multiple channel laser emission enhances the laser target echo signal amplitude and also improves the anti-interference ability against the combined effects of multiple particle features compared with the single channel. Through the validation based on the laser prototype with four-channel beam emitting, the above conclusions are supported by the experimental results. Therefore, this study not only reveals the laser target properties under the 3D particle collision perspective, but also reflects the reasonableness and effectiveness of utilizing the target characteristics in the 3D particle collision mode to enhance the detection performance of FMCW laser fuze in the smoke.

## 1. Introduction

As a novel active fuze that applies laser technology to the proximity fuze, the laser proximity fuze can detonate weapon explosives at the right time and space by controlling the laser’s ranging performance with high precision [1]. In the smoke, the internal particles are mostly generated in situations such as fuel combustion and explosions. Compared to sand, rain and snow, smoke particles with smaller particle sizes are suspended in the atmosphere for longer periods of time, and the laser fuze for the ground target is interfered with by the smoke-suspended particles at low-altitude detection, which causes laser energy attenuation and spatial distribution distortion, resulting in the false alarms and faults [2]. In particular, frequency-modulated continuous-wave (FMCW) laser detection technology uses beat information to analyze the echo characteristics and extract the target distance and other information; compared with pulsed laser detection in dense smoke conditions, it has better anti-interference performance [3]. However, the target detection and identification of FMCW laser fuzes are still challenging when considering the complex effects of multiple laser backscattering caused by smoke particles.

To improve the detection performance and anti-interference capability of FMCW laser fuze in the smoke environment, it is necessary to study the laser echo signal model and target characteristics based on multiple backscattering. Under different smoke visibility conditions, the target frequency domain characteristics and their variation patterns can be extracted [4]. Regarding the study of target echo characteristics of laser fuze detection in the aerosol environment, scholars currently focus on building laser backscattered and attenuation models based on multipath transmission effects to research target characteristics [5,6]. Based on Monte-Carlo method, Zhang investigates the features of smoke echo signals at different distances and smoke visibilities, and the results show that smoke causes the laser multipath transmission effect which leads to the spread of the beat signal spectrum [7]. Liu simulates the variation of SNR between the particle size of smoke particles and the laser wavelength, and the results show that larger wavelengths are more likely to cause laser multiple scattering, which leads to the reduction of SNR [8]. Wang compares the differences in the amplitude of near-, mid-, and far-infrared laser echoes at different visibilities, and the results show that the laser wavelength is negatively correlated with the amplitude of echo signals, where the decrease in target distance leads to the laser echo spreading [9]. Pang analyzes the amplitude variation of laser echo signals with different laser wavelengths in four aerosol environments, and the results show that the target distance is related to the scattering echoes of aerosol particles, but not to the target echoes [10].

In this approach, the optical range and scattering direction of photons are sampled using one-dimensional (1D) random sampling to simulate the two-dimensional (2D) motion process of photons in the smoke, which weakens the combined effect of smoke particle properties, spatial location and physical characteristics during the process [11]. Thus, it means ignoring the uncertainty in spatial position distribution caused by the temporal and spatial variations of smoke particles inside the scene [12]. When all photons move toward the interior of smoke particle environment, it is difficult to reflect the target characteristics and changes caused by the combined effects of different particle features. For the three-dimensional (3D) collision process between photons and smoke particles, the laser echo signal model and target characteristics based on the 2D collision mode have limitations.

The root cause of the above situation is the difference in realism between 2D and 3D collision simulations, and the laser target properties extracted by the former are difficult to reflect the temporal and spatial properties under 3D particle collisions. Thus, it is hard to analyze the influence of combined effect on the laser target characteristics of photon transmission and smoke particle features, which is detrimental to further improvement in the detection performance of FMCW laser fuzes in the smoke.

To solve these problems, it is necessary to build a laser echo signal model based on 3D particle collisions. By conducting laser echo signal simulations that embody the temporal and spatial properties of 3D particles, the target characteristics under the combined effect of 3D particle features can be output and extracted. On this basis, considering that increasing the laser emission power and improving the laser emission structure can effectively enhance the amplitude of target echo signal and improve the signal-to-noise ratio (SNR), in-depth research on the techniques of anti-smoke interference, target echo enhancement and fuse detector optimization can be achieved by studying the combined effect of the multiple channel laser emission structure on the 3D particle features, which can effectively enhance the detection performance of the FMCW laser fuzes in the smoke environment.

Therefore, the 3D collision and transmission process of photons in the smoke environment is studied in Section 2, and the modeling of FMCW laser echo signal based on 3D particle collision and scattering is established in Section 3. Through simulating the effect of photon transmission process and smoke particle features on the laser echo signal based on the model from Section 3, the combined effect of multiple laser emission on the particle characteristics is researched in Section 4. Based on this, the design and utilization of the detection system based on the multiple channel laser emission structure is experimentally verified in Section 5. Conclusions are summarized in Section 6.

## 2. Three-Dimensional Paritcle Collision and Transmission Process

As quanta of electromagnetic radiation, photons have no rest mass, whereas smoke particles have mass. This mass difference leads to the completely different dynamic behavior of the two in collision process. The accurate process of photon scattering needs to be described considering the theories of quantum electrodynamics [13]. However, photons have momentum, and their scattering direction depends on the interaction with smoke particles. Although the movement direction of photon changes, the total momentum is conserved during the scattering process. Thus, to obtain the scattering direction of photons, the collision of photons with smoke particles can be simplified to a perfectly elastic collision between particles with shapes based on wave–particle duality and collision dynamics. The photon particle in subsequent contents are not actual physical photons in quantum electrodynamics [14].

For the 3D particle collision process between photons and smoke particles, it can theoretically be considered as a mixed system containing internal collisions of the same particle systems and external collisions of different particle systems. At the moment when laser fuze detector turns on, the speed of light is far larger than the speed of smoke particles under external forces, so it can be considered that smoke particles are stationary relative to photons. At the same time, considering that the photon composition system does not follow the Pauli exclusion principle, it can ignore the internal collision process of photons and smoke particles. Thus, it is only necessary to focus on the particle collision process between the different systems, which are the mutual collision process in the particle systems of photons and smoke particles.

**Figure 1:**
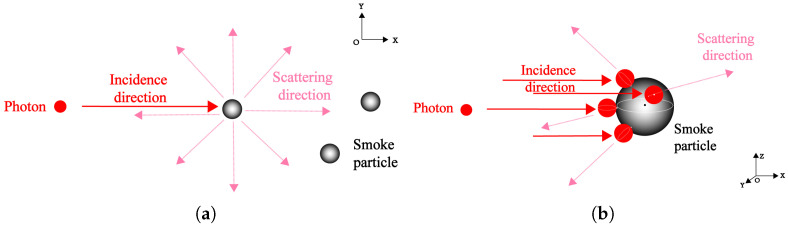
Two ways of photon collision with smoke particle based on the sphere: (**a**) 2D collision ignoring the particle space shape, (**b**) 3D collision approach existing with the particle space shape.

Laser fuzes are typically used in the laser wavelength range of 0.65 μm to 1.65 μm, and the majority of smoke particles are in the size range of 1 μm to 10 μm. The size parameter of the two particles is relatively close. Therefore, the scattering process between photons and smoke particles needs to be described by the Mie scattering model. Under spherical particle collisions, two ways of photon collision with a smoke particle are shown in Figure 1.

**Figure 2:**
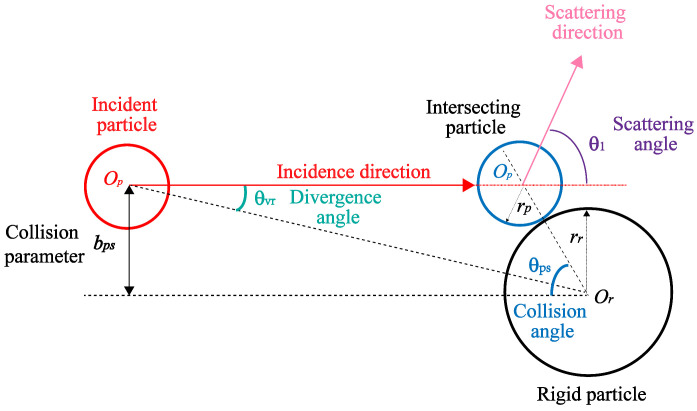
Single scattering angle $\theta_{1}$, collision angle $\theta_{ps}$, velocity deflection angle $\theta_{vr}$ and collision parameter $b_{ps}$ based on rigid-body hard sphere scattering.

**Figure 3:**
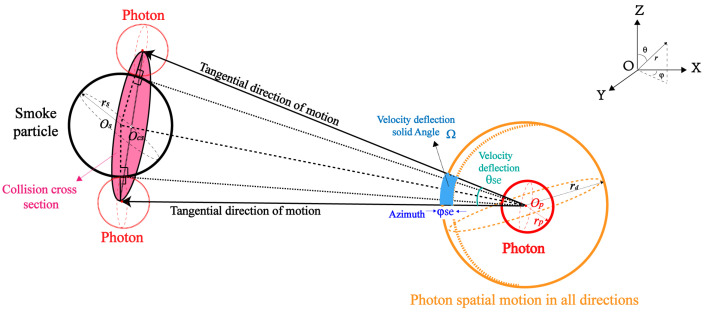
Maximum velocity deflection solid angle Ω, azimuth $\varphi_{se}$ and collision cross-section for 3D collisions of photons in smoke environments.

Under the conditions of elastic collision and scattering, the process of photon scattering by smoke particles can be regarded as the collision motion process of incident particles, which is shown in Figure 2. The collision parameter $b_{ps}$ is the vertical distance between the direction of particle incidence and center of the rigid-body particle. The 3D particle collision detection results can be obtained through the particle motion tracks and the intersection in two consecutive time steps. When particle collision occurs, the maximum velocity deflection solid angle is shown in Figure 3. At this time, the collision conditions of photons and smoke particles are determined by collision probability.

From Figure 1b, it can be seen that the direction of particle motion is deflected after the collision occurs. As a cone representation of a solid angle, the maximum velocity deflection angle Ω is axisymmetric based on the line connecting the center of the sphere. It can be considered that the collision cross-section is independent of the azimuth $\varphi_{se}$, and the integration range of $\varphi_{se}$ can be set as [0,2π]. The collision cross-section at unit solid angle $\varsigma_{ps}\left(\theta_{1}\right)$ is the ratio of collision section d$\sigma_{ps}$ to velocity deflection angle $d\Omega_{vr}$, which can be expressed in Equation (1).

(1) $$\varsigma_{ps}\left(\theta_{1}\right)=\frac{d\sigma_{ps}}{d\Omega_{vr}}=\frac{b_{ps}db_{ps}d\varphi }{\sin\theta_{1}d\theta_{1}d\varphi }=\frac{b_{ps}}{\sin\theta_{1}}\times \left|\frac{db_{ps}}{d\theta_{1}}\right|$$

where $d\sigma_{ps}$ is the collision cross-section for $\theta_{1}$ on $\left[\theta_{1},\theta_{1}+d\theta \right]$, $d\Omega_{vr}$ is the solid angle on $\left[\Omega_{vr},\Omega_{vr}+d\Omega \right]$, and total collision cross-section $\sigma_{ps}$ can be expressed as

(2) $$\sigma_{ps}=\int_{0}^{4\pi }\varsigma_{ps}\left(\theta_{1}\right)d\Omega =\pi {\left(r_{s}+r_{p}\right)}^{2}$$

where $r_{p}$ and $r_{s}$ are the size of photon and smoke particle, respectively. The collision probability between photons and smoke particles can be calculated as

(3) $$p_{sc}\left(t\to t+\Delta t\right)=\sum_{i=1}^{n_{smoke}}\frac{n_{s_{grid}}}{n_{smoke}}\times \pi {\left(r_{s}+r_{p}\right)}^{2}\times \Delta v_{ps}^{\left(i\right)}\Delta t$$

where $n_{s_{grid}}$ and $n_{smoke}$ are the number of smoke particles in the localized area and the total number in the scene, respectively, $\Delta v_{ps}$ is the relative velocity of the incident particle, Δt is the time step.

Thus, the weight coefficient is used to characterize the photon energy changes. The initial weight $W_{0}$ is set to one, and the energy weights of photons after multiple collisions with smoke particles can be expressed as

(4) $$W_{n_{sca}}=\prod_{n_{sca}=1}^{n_{c_{max}}}p_{sc}^{\left(n_{sca}\right)}\times \eta_{s}^{\left(n_{sca}\right)}\times W_{0}$$

where $\eta_{s}$ is the energy attenuation coefficient based on Mie scattering, $n_{sca}$ and $n_{c_{max}}$ are the scattering number and maximum number of collisions.

**Figure 4:**
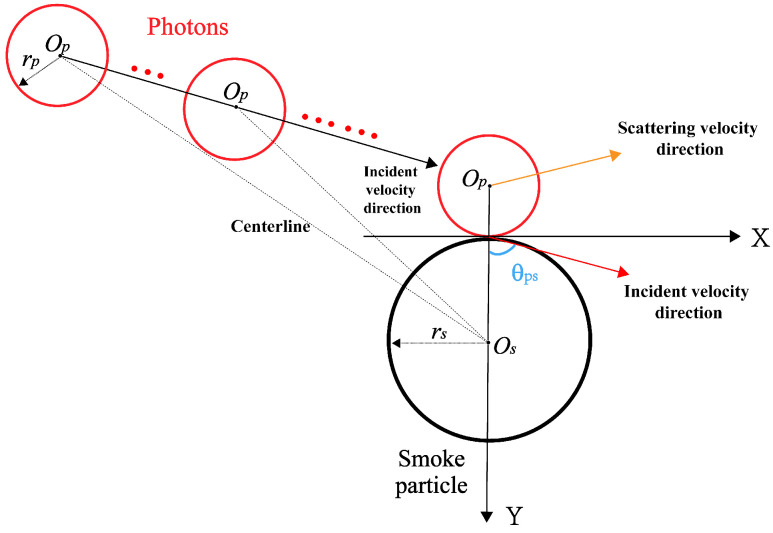
The variation of velocity vector in the non-centric collision mode.

For the 3D particle collision, it is a mixed situation combining centric and non-centric modes from Figure 1b. As the velocity of smoke particles $v_{s}$ can be regarded as 0, it shows that the 3D non-centric collision process can be simplified to a 2D form. Photon and smoke particles need to be smooth to avoid meaningless rotation during collision. The velocity vector in the non-centric collision mode is shown in Figure 4.

In the interaction between mesoscopic particles and photons, the momentum of a single photon is too small to make the momentum of a mesoscopic particle change significantly. Considering the momentum fluctuation of a single photon, it cannot be directly assumed that the momentum conservation between a single photon and a mesoscopic particle is strict. However, the momentum of photon tends to a stable value based on the average effect of a large number of particles in the same path, which forms a conservation relationship with the momentum of mesoscopic particles. Thus, the system momentum is constant before and after the collision, which can be concluded as

(5) $$p_{p}+p_{s}=p_{p}^{\prime }+p_{s}^{\prime }$$

where $p_{p}$ and $p_{s}$ are the momentum of photon particle and smoke particle before collision occurrence, respectively, where the former is expressed as $p_{p}=\frac{hv}{c}$ and the latter can be set as $p_{s}=0$. When collision occurs, the photon particle momentum and the smoke particle momentum change to $p_{p}^{\prime }$ and $p_{s}^{\prime }$, respectively.

Under conditions of perfectly elastic collision without other external forces, the collision direction of photons can be expressed by the change in particle momentum vectors before and after the collision. Based on Equation (5), the sum of momentum vectors is also constant and it can be concluded as

(6) $${\vec{p}}_{p}+{\vec{p}}_{s}={\vec{p}}_{p}^{\prime }+{\vec{p}}_{s}^{\prime }$$

where ${\vec{p}}_{p}$ and ${\vec{p}}_{p}^{\prime }$ are the momentum vectors of photon particles before and after collision, respectively, and the angle between ${\vec{p}}_{p}$ and ${\vec{p}}_{p}^{\prime }$ is expressed as the scattering angle $\theta_{1}$. Thus, according to Figure 2 and Figure 4, $\theta_{1}$ can be expressed by quantifying the absolute deflection degree of the collision as

(7) $$\theta_{1}=\left|\pi -2\theta_{ps}\right|=2\arccos\frac{b_{ps}}{r_{s}+r_{p}}$$

In summary, using the equations for scattering direction and energy, it is possible to describe the collision and transmission process of photons in the smoke environment, which provides the basis for research on modeling of laser echo signals.

## 3. Modeling of FMCW Laser Echo Signal

**Figure 5:**
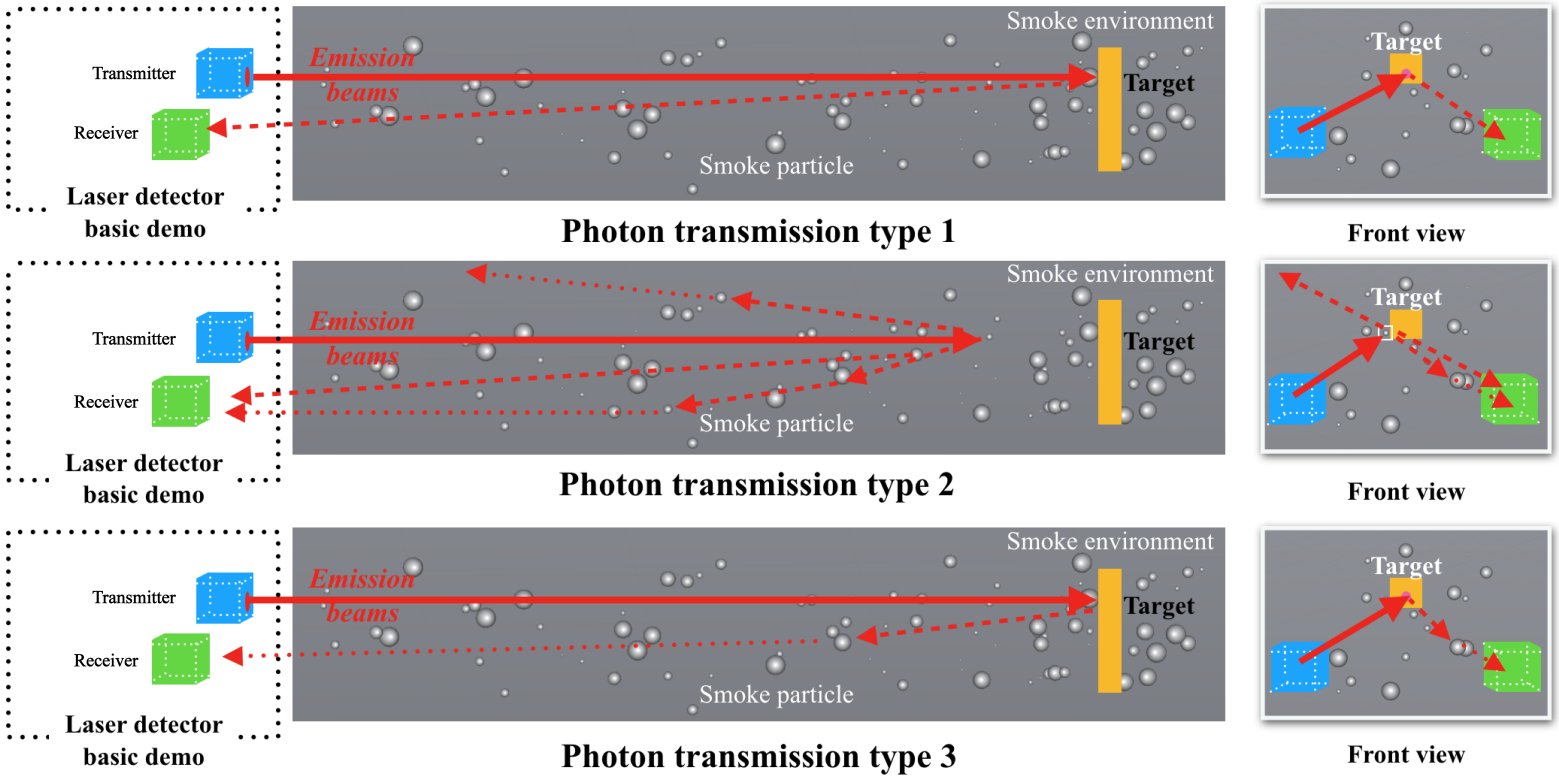
Photon motion process based on three types in smoke environment.

The schematic diagram of photon transmission in a spatial environment filled with smoke particles can be seen in Figure 5, which shows that multiple paths exist in the process of photons from emission to reception. In particular, in view of the low operating height of the laser fuze to the ground and diffuse reflection from the target, the photon transmission process can be further summarized into three main types. The corresponding laser echo power signals are expressed as $S_{R{\_}_{tar}}\left(t\right)$, $S_{R{\_}_{smoke}}\left(t\right)$ and $S_{R{\_}_{blend}\left(t\right)}$.

Under these conditions, based on Equation (4) for weighting in multiple scattering and Equation (7) for the relationship between scattering angle $\theta_{1}$ and collision angle $\theta_{ps}$, scattering angle $\theta_{1}$ can be characterized by $\theta_{ps}$ of the 3D collision mode, and the laser echo power signals are described as follows for each type of photon transmission.

(1) The photons are only reflected by the target and received directly by the detector.

For the laser detection system, the laser photons under such conditions are mainly scattered and absorbed by atmospheric particles. Thus, based on the laser radar distance equation [2], the illumination of laser beam under Gaussian distribution [15] and scattering cross-section equation for the laser illumination area [16], $S_{R{\_}_{tar}}\left(t\right)$ can be expressed in Equation (8),

(8) $$S_{R{\_}_{tar}}\left(t\right)=\frac{P_{T1}A_{r}\eta_{atm}\eta_{sys}\rho_{T}\cos\theta_{inc}}{R^{2}}e^{-2\sigma_{ext}R}\times S_{T}\left(t-\tau_{T}\right)$$

where $A_{r}$ is the laser receiving optical area; $\eta_{atm}$ and $\eta_{sys}$ are the atmospheric and optical transmittance, respectively, which can be considered approximately constant at short range detection; $\rho_{T}$ is reflectivity of the ground extended target; $\theta_{inc}$ is the angle of incidence, which is equal to $\theta_{ps}$ based on Figure 2; $P_{T1}$, $S_{T}$ and $\tau_{T}$ are the laser emission power, laser emission power signal and time delay in this situation.

(2) The photons are scattered by the smoke particles and received by the detector.

Under such condition, considering the uncertainty of local spatial position distribution for smoke particles, laser photons appear to be scattered by smoke boundary particles in both single backscattering and multiple scattering. According to the multiply scattering model [17], the echo power can be expressed as

(9) $$P_{\delta_{v_{i}}}=\frac{Q_{sca}}{2\left(1-\cos\frac{1}{2}\theta_{T}\right)l_{i}^{2}}p\left(\theta_{s_{i}}\right)e^{-Q_{ext}l_{i}}P_{\delta_{v_{i}-1}}\delta_{v_{i}}$$

where $\delta_{v_{i}}$ and $\delta_{v_{i}-1}$ are the unit space volume of the scattered photon under continuous scattering conditions; $P_{\delta_{v_{i}}}$ and $P_{\delta_{v_{i}-1}}$ are the scattered power of $\delta_{v_{i}}$ and $\delta_{v_{i}-1}$, respectively; $\theta_{T}$ is the laser beam divergence angle; $l_{i}$ is the movement distance of the photon; $Q_{sca}$ and $Q_{ext}$ are the scattering and extinction factor [18], respectively; and $p(\theta_{s_{i}})$ is the scattering phase function based on the Mie scattering model, which reflects the distribution of light scattering energy by aerosol particles [19]. Thus, $p(\theta_{s_{i}})$ is only related to $\theta_{1}$. According to Equation (7), $p(\theta_{s_{i}})$ can be expressed as

(10) $$p\left(\theta_{s_{i}}\right)=p\left(\theta_{1}\right)=p\left(\left|\pi -2\theta_{ps}\right|\right)$$

When the photon is received by the detector, the laser echo power in this situation can be expressed as

(11) $$\begin{array}{cc} S_{R{\_}_{smoke}}\left(t\right)=\int \cdots \int_{v_{1}\cdots v_{M}} & P_{T2}\frac{A_{r}\eta_{atm}\eta_{sys}Q_{sca}^{M}\cos\varphi_{rc}}{2(1-\cos\frac{1}{2}\theta_{T})}\times \prod_{i=1}^{M}\frac{p(\left|\pi -2\theta_{ps_{i}}\right|)}{l_{i}^{2}}\\ \; & \times e^{\left(-\sigma_{ext}\times \sum_{i=1}^{M}l_{i}\right)}\delta_{v_{1}}\cdots \delta_{v_{M}}\times S_{T}\left(t-\tau_{M}\right) \end{array}$$

where $P_{T2}$ and $\tau_{M}$ are laser emission power and time delay in this situation, respectively, and $\varphi_{rc}$ is the angle between received light and the axis of the received field of view (FOV). $\theta_{ps_{i}}$ is scattering phase function corresponding to $\theta_{ps}$ at a scattering number of *i*.

(3) The photons are scattered by multiple particle collisions and received by detectors.

The collision scattering of photons in complex smoke scenes is affected by the type of target. The laser echo power signal is expressed as

(12) $$\begin{array}{cc} S_{R{\_}_{blend}\left(t\right)}=\int \cdots \int_{v_{1}\cdots v_{N}} & P_{T3}\frac{A_{r}\eta_{atm}\eta_{sys}Q_{sca}^{M}\cos\varphi_{rc}}{2(1-\cos\frac{1}{2}\theta_{T})}\times \prod_{j=1}^{N}\frac{p(\left|\pi -2\theta_{ps_{j}}\right|)}{l_{j}^{2}}\\ \; & \times \rho_{T}^{n_{T}}\cos^{n_{T}}\psi_{T}\prod_{Bi=1}^{B_{max}}\rho_{Bi}^{n_{Bi}}\cos^{n_{Bi}}\psi_{Bi}\\ \; & \times e^{\left(-\sigma_{ext}\cdot \sum_{j=1}^{N}l_{j}\right)}\delta_{v_{1}}\cdots \delta_{v_{N}}\times S_{T}\left(t-\tau_{N}\right) \end{array}$$

where $P_{T3}$ and $\tau_{N}$ are laser emission power and time delay in this situation, respectively; Bi is the types of object; $\psi_{T}$ and $\psi_{Bi}$ are the incidence angle of photons with ground target and other scatterers, respectively, corresponding to the number of collisions $n_{T}$ and $n_{Bi}$; $\rho_{Bi}$ is the scattering coefficient of these scatterers; $\theta_{ps_{j}}$ is scattering phase function corresponding to the $\theta_{ps}$ at a scattering number of *j*.

According to Equations (8), (11) and (12), the laser echo power signal is summarized as

(13) $$S_{R}\left(t\right)=\sum_{i=1}^{n_{p1}}S_{R{\_}_{tar}}\left(t\right)+\sum_{j=1}^{n_{p2}}S_{R{\_}_{smoke}}\left(t\right)+\sum_{k=1}^{n_{p3}}S_{R{\_}_{blend}\left(t\right)}$$

where $n_{p1}$, $n_{p2}$ and $n_{p3}$ are the actual numbers of photons received.

**Figure 6:**
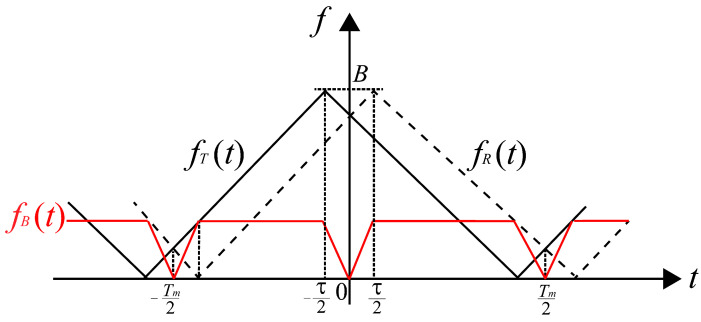
FMCW detection principle based on linear FM system with triangular wave.

Through the processing of the beat signal, the frequency domain characteristics of the target echo signal and smoke echo signal and its changing law can be extracted. At this time, the target characteristics of the laser fuze reflect the time and space attributes of the 3D particle collision. The beat signal $S_{B}\left(t\right)$ can be expressed as

(14) $$S_{B}\left(t\right)=S_{T}\left(t\right)\times S_{R}\left(t\right)+S_{N}\left(t\right)$$

where $S_{N}\left(t\right)$ is the random noise signal and the spectrum of $S_{B}\left(t\right)$ can be obtained by fast Fourier Transform (FFT). FMCW detection principle based on triangle wave frequency modulation can be seen in Figure 6, where $f_{T}\left(t\right)$, $f_{R}\left(t\right)$, $f_{B}\left(t\right)$ are the instantaneous frequency of transmission signal, target echo signal and beat signal and *B*, $T_{m}$ and τ are the sweep bandwidth, modulation period and time delay, respectively. The horizontal and vertical axes in Figure 6 are time and frequency, respectively. The laser echo signal is obtained after a time delay of the transmission signal, and the frequency of the echo signal can be considered as the translation of transmission signal in time, as shown by the dashed line in Figure 6.

The working distance of laser fuzes is usually in the range of a dozen to tens of meters, and time delay τ is in the nanosecond range, which can be regarded as $\tau \ll T_{m}$. The beat signal can be considered as a periodic signal, as shown by the red solid line in Figure 6, which can be expressed as

(15) $$f_{B}\left(t\right)=f_{B}\left(t-\frac{T_{m}}{2}\right)=\left|f_{R}\left(t\right)-f_{T}\left(t\right)\right|=\left\{\begin{array}{ccc} \; & \frac{4B}{T_{m}}t, & 0⩽t⩽\frac{\tau }{2}\\ \; & \frac{2B}{T_{m}}\tau , & \frac{\tau }{2}⩽t⩽\frac{T_{m}}{2}-\frac{\tau }{2}\\ \; & -\frac{4B}{T_{m}}t+2B, & \frac{T_{m}}{2}-\frac{\tau }{2}⩽t⩽\frac{T_{m}}{2} \end{array}\right.$$

Under the condition of triangular wave linear frequency modulation signals, time delay τ is $\frac{2R}{c}$ and beat frequency $f_{B}$ is $\frac{2B}{T_{m}}\tau$, where *c* is the speed of light. Thus, detection distance *R* is expressed as

(16) $$R=\frac{cT_{m}}{4B}f_{B}$$

**Figure 7:**
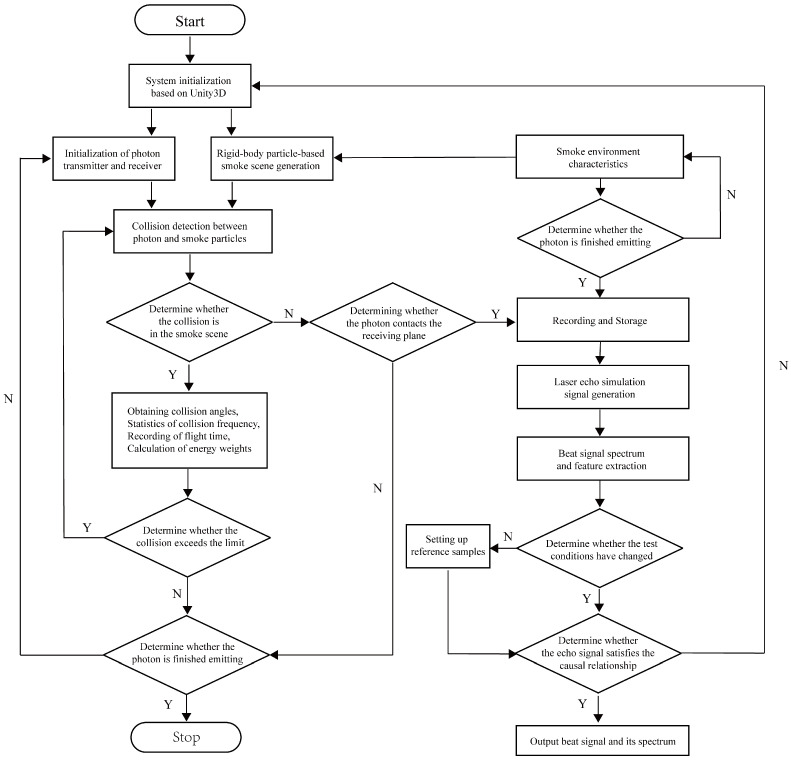
Simulation process of FMCW laser beat signal based on 3D particle dynamic collision.

Furthermore, the model of Equation (13) contains several parameters and variables such as collision angle, which is difficult to directly extract and analyze the target characteristics. At this time, to obtain $\theta_{ps}$ in the 3D collision process, it is necessary to use the virtual particle system simulation based on the Monte-Carlo method in Unity3D, which has a powerful physics engine [20], a professional particle system [21] and a multi-system combined simulation capability [22]. This approach has also been proven to be reasonable and effective for FMCW laser’s target echo characteristic simulation in [23]. Depending on the amplitude of $S_{T}\left(t\right)$, the number of particle emission models $n_{p}\left(t\right)$ can be set as $n_{p}\left(t\right)=\eta_{p}S_{T}\left(t\right)$, where $\eta_{p}$ is the conversion factor. Considering that the smoke environment is a mixture of soot and organic particles with sizes in the range of a few micrometers to more than a dozen micrometers, its influence on the laser detection performance is not significant to set the same size of smoke particles [24]. The main simulation parameters are shown in Table 1, and the process is shown in Figure 7. On this basis, the laser echo simulation signal and beat signal can be output.

**Figure 8:**
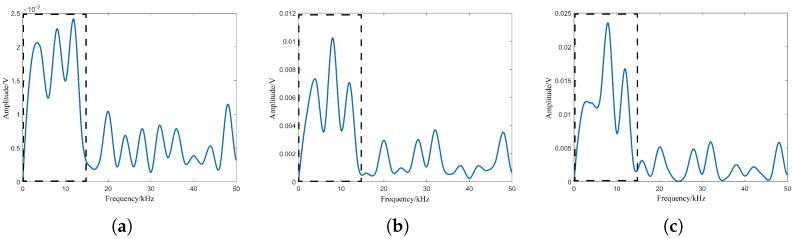
Laser beat signal spectrums with different features of photon transmission: (**a**) Emission number, (**b**) divergence angle, (**c**) receiving field of view.

**Figure 9:**
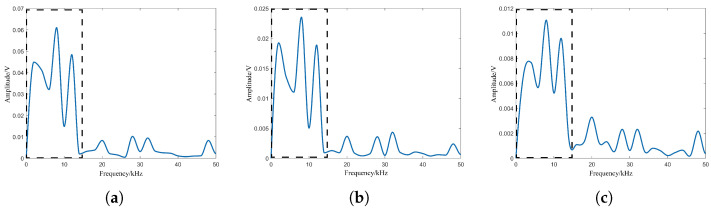
Laser beat signal spectrums with different features of smoke particles: (**a**) Number, (**b**) size, (**c**) height position.

Considering the difference in performance between the transmitter and the receiver of the laser fuze detector and the difference in particle characteristics within the smoke, laser echo signal simulation is needed here for the photon transmission and the smoke particle features. Among them, the photon transmission features include the emission number, the divergence angle and the receiving field of view, and the smoke particle features include the number, the particle size and the height position. The simulation results are shown in Figure 8 and Figure 9 based on the consistent smoke visibility, respectively. The target position corresponds to a beat frequency of 12 kHz based on Equation (16) and the dashed box indicates the waveform with frequency 0–15 kHz.

In 3D particle collision mode, it can be seen that the waveforms in the dashed box are completely different. For the photon transmission feature, the effect of emission number on the amplitude of target echo signal is relatively larger: the SNR is 0.6 dB. The SNRs for the other two features are less than 0. For the smoke particle feature, the waveforms of smoke particle number and particle size in the dashed box are similar, and their SNRs are −2.3 dB and −2.2 dB, respectively. However, the SNR of height position is −1.4 dB, which shows that the effect of smoke particle position on the amplitude of target echo signal is relatively greater. Therefore, the 3D particle collision model needs to focus more on the effects of the photon emission number and smoke particle position on the laser echo signal and its individual components, and their impact on the amplitude of the target echo signal needs to be further studied and analyzed.

## 4. Simulation of Laser Target Characteristics

### 4.1. Multiple Channel Laser Emission Structure

**Figure 10:**
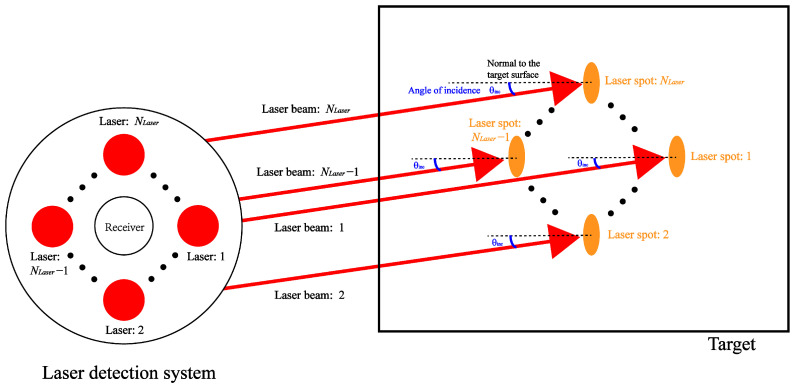
The structure and detection process based on multiple channel laser emission.

In the perspective of 3D particle collision, to deal with the significant impact of the laser power and the spatial position of smoke particles on the target characteristics, the amplitude of target echo signal can be effectively enhanced by increasing the laser power and improving the laser structure so as to improve the SNR. Under this condition, the structure and detection process based on multiple channel laser emission is shown in Figure 10.

At this time, by providing a plurality of lasers, it is possible to equivalently increase the overall power of the emitted laser. Moreover, by adopting a symmetrical structure, the lasers can be uniformly distributed around the receiver, and the influence caused by the spatial position of the smoke particles can be reduced. On the basis of the above two ways, the increase in the amplitude of the target echo signal can be achieved. To make it easier to analyze and calculate, individual lasers can be set at the same distance from each other. According to Equation (8), the echo signal of the multiple channel laser emission condition under smoke-free conditions can be expressed as

(17) $$\begin{array}{cc} S_{R\_MultiLaser}\left(t\right)= & \frac{P_{T1}A_{r}\eta_{atm}\eta_{sys}\rho_{T}\cos\theta_{inc\_1}}{R_{1}^{2}}e^{-2\sigma_{ext}R_{1}}S_{T}\left(t-\tau_{1}\right)+\cdots \\ \; & +\frac{P_{T1}A_{r}\eta_{atm}\eta_{sys}\rho_{T}\cos\theta_{inc\_N_{Laser}}}{R_{N_{Laser}}^{2}}e^{-2\sigma_{ext}R_{N_{Laser}}}S_{T}\left(t-\tau_{N_{Laser}}\right) \end{array}$$

According to Figure 3, the collision probability is related to the smoke particles in the local area and the collision cross-section. When the number of optical paths is changed from single channel to multiple channels, the collision probability can be expressed as

(18) $$\begin{array}{c} p_{scN}\left(t\to t+\Delta t\right)=\sum_{j=1}^{N_{Laser}}\sum_{i=1}^{n_{smoke}}\frac{n_{s_{grid\_j}}}{n_{smoke}}\times \pi {\left(r_{s}+r_{p}\right)}^{2}\times \Delta v_{ps}^{\left(i\right)}\Delta t \end{array}$$

According to collision probability $p_{sc}$ under single optical channel conditions, the result of their ratio $\eta_{low}$ in low visibilities can be expressed as

(19) $$\eta_{low}=\frac{p_{sc}\left(t\to t+\Delta t\right)}{p_{scN}\left(t\to t+\Delta t\right)}=1-\frac{\sum_{j=2}^{N_{Laser}}\sum_{i=1}^{n_{smoke}}\frac{n_{s_{grid\_j}}}{n_{smoke}}\times \pi {\left(r_{s}+r_{p}\right)}^{2}\times \Delta v_{ps}^{\left(i\right)}\Delta t}{\sum_{j=1}^{N_{Laser}}\sum_{i=1}^{n_{smoke}}\frac{n_{s_{grid\_j}}}{n_{smoke}}\times \pi {\left(r_{s}+r_{p}\right)}^{2}\times \Delta v_{ps}^{\left(i\right)}\Delta t}<1$$

According to Equation (7), when the single scattering angle exists for the emitted photons of any channel in high visibilities, other optical channels of the emitted photons do not exist with a single scattering angle. Thus, $n_{s\_grid}=n_{s\_grid\_1}\approx n_{s\_grid\_2}\approx \cdots \approx n_{s\_grid\_N_{Laser}}$, and the collision probability is expressed as

(20) $$\eta_{high}=\frac{p_{sc}\left(t\to t+\Delta t\right)}{p_{scN}\left(t\to t+\Delta t\right)}=\frac{\sum_{i=1}^{n_{smoke}}\frac{n_{s_{grid}}}{n_{smoke}}\times \pi {\left(r_{s}+r_{p}\right)}^{2}\times \Delta v_{ps}^{\left(i\right)}\Delta t}{\left(N_{Laser}-1\right)\times 0+\sum_{i=1}^{n_{smoke}}\frac{n_{s_{grid\_N_{Laser}}}}{n_{smoke}}\times \pi {\left(r_{s}+r_{p}\right)}^{2}\times \Delta v_{ps}^{\left(i\right)}\Delta t}⩾1$$

In low visibility, the multiple optical channels cause an increase in the probability of collision, and in medium and high visibility, multiple optical channels reduce the probability of collision. Thus, the above situations demonstrate that it is indeed theoretically feasible to adapt a single optical channel to a multi-channel structure based on the 3D particle collision process.

### 4.2. Particle Feature Co-Simulation Results

According to Figure 10, the results of multi-channel beam simulation can be output through the superposition of a single channel. In this section, the visibility of the smoke environment is set to 12 m and 15 m, and the type of particle joint feature is between the number of photons emitted and the height position of the smoke particles. In this section, the laser target characteristics are simulated and compared with single- and four-channel laser emission structures, where the simulation parameters are set based on Table 1.

(1) Co-simulation of particle features in the single-channel laser emission structure.

**Figure 11:**
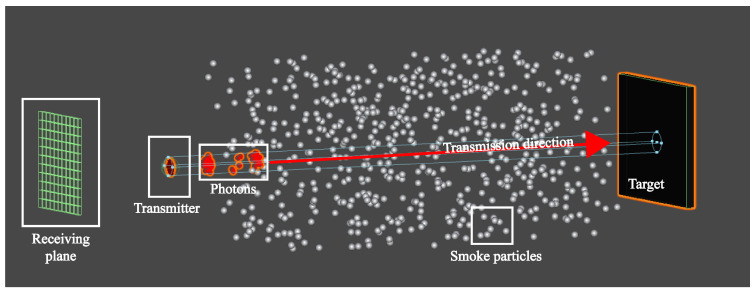
The main transmission process of photon in the single-channel emission structure.

**Figure 12:**
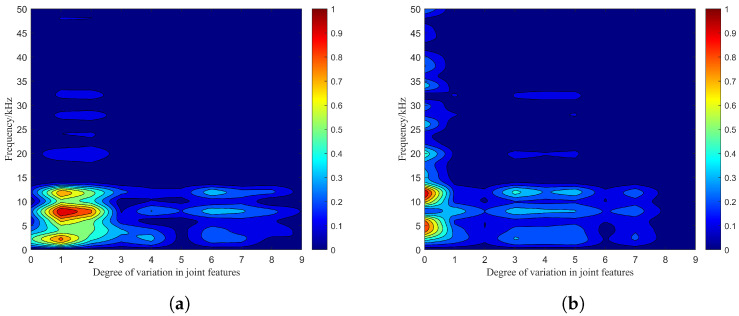
Amplitude–frequency characteristics of the beat signal spectrum in the single-channel emission structure: (**a**) Amplitude–frequency characteristics for a visibility of 12 m, (**b**) amplitude–frequency characteristics for a visibility of 15 m.

In the virtual environment of Unity3D, the main process of photon emission and reception is shown in Figure 11, and the combined effect of particle features is divided into 10 types. These types can be respectively notated as 0–9 according to the degree of difference, where type 0 indicates no effect and type 9 indicates maximum effect. The single feature difference value is set from 0% to 90% of the initial value, and the joint features can be set simultaneously based on single feature values. The receiving plane consists of unit planes based on the receiving field of view. For the target, amplitude–frequency characteristics of the beat signal spectrum are shown in Figure 12.

It can be seen that the combination of the photon emission number and the smoke particle position characteristics causes fluctuations in the maximum peak of the beat signal spectrum. At this point, according to the bijection relationship between beat frequency and distance in Equtaion (16), the target detection distance corresponding to the beat frequency at the maximum peak changes uncertainly. In this case, the initial frequency is 3 kHz, the average value of the maximum peak frequency is 8 kHz at 12 m visibility, and the initial frequency is 12 kHz and the average value of the maximum peak frequency is 9 kHz at 15 m visibility. Compared to the single-feature case in Figure 8a and Figure 9c, the combination of particle features results in the expansion of the peak fluctuation area and number, which means that the combined effect of the particle features exacerbates the effect on the laser echo signal and the individual components.

(2) Co-simulation of particle features in the four-channel laser emission structure.

**Figure 13:**
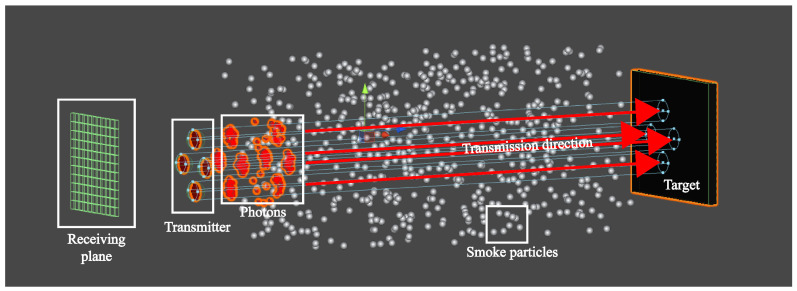
The main transmission process of photon in the four-channel emission structure.

**Figure 14:**
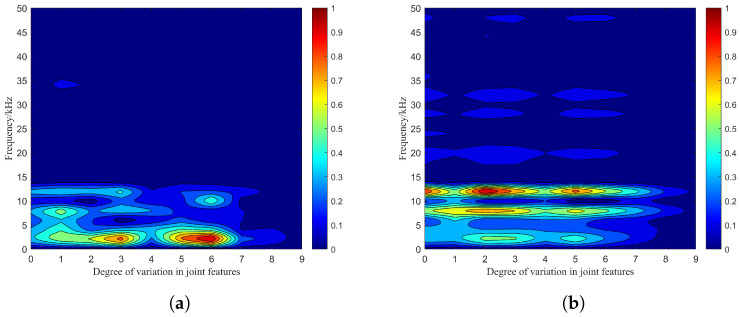
Amplitude–frequency characteristics of the beat signal spectrum in the four-channel emission structure: (**a**) Amplitude–frequency characteristics for a visibility of 12 m, (**b**) amplitude–frequency characteristics for a visibility of 15 m.

In the virtual environment of Unity3D, the main process of photon emission and reception is shown in Figure 13. According to the way of single-channel emission, the co-simulation of particle features under the four-channel emission is performed, where the emission delay between each optical path is neglected. The amplitude–frequency characteristics of the beat signal spectrum are shown in Figure 14.

It can be seen that the initial frequency is 3 kHz and the average value of the maximum peak frequency is 3 kHz at a visibility of 12 m. The initial frequency is 12 kHz and the average value of the maximum peak frequency is 12 kHz at a visibility of 15 m. Compared with the effect of a single-channel emission structure on the combined effect of the particle features, the multiple-channel emission structure causes the reduction in the peak fluctuating area and number. At this time, the negative impact of the combined effect of particle features on the laser echo signal and the individual components are reduced to some extent.

From Figure 14b, it can be seen that the maximum peak of the spectrum of the beat signal is basically stabilized at 12 kHz under the visibility condition of 15 m. According to Equation (16) with the parameters in Table 1, the distance corresponding to this peak frequency can be calculated as

(21) $$R=\frac{cT_{m}}{4B}f_{B}=\frac{3\times 10^{8}\;m/s\times 0.5\;\mathrm{ms}}{4\times 150\;\mathrm{MHz}}\times 12\;\mathrm{kHz}=3\;m$$

Based on Equation (21), the result is consistent with the preset target distance, which means that the maximum peak of the spectral spectrum of the beat signal and the spectrum peak of the target’s echo signal component are the same under this visibility condition. By comparing the similarity of target characteristics, the impact of combined effect of the particle characteristics can be further analyzed.

### 4.3. Evaluation of Simulation Similarity and Comparison Analysis

The similarity degree of particle feature combined effect simulation can be calculated by comparing the beat signal spectrum peaks under single channel and four channels. In this case, the lower the degree of similarity, the more significant the impact of the particle feature combined effect, which is expressed in Equation (22).

(22) $$\eta_{ss}=\frac{n_{similarity}}{n_{Ts}}$$

where $n_{similarity}$ and $n_{Ts}$ are the number of peak frequencies that are the same and the number of combined effect types, respectively.

**Figure 15:**
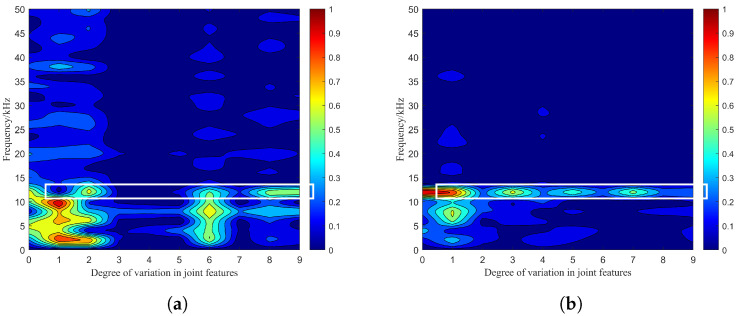
Amplitude–frequency characteristics of the beat signal spectrum based on the target echo signal components at visibility conditions of 15 m: (**a**) Amplitude–frequency characteristics in a single-transmission structure, (**b**) amplitude–frequency characteristics in four -transmission structure.

Therefore, through the photon tracking of the target plane, the virtual simulation flow of Figure 7 is able to output the target echo signal components of the mixed echo signals under the single and four-channels structures, and the corresponding amplitude–frequency characteristics are shown in Figure 15. The average simulation similarities are 0.44 and 0.89, and the increase is about 20%.

**Figure 16:**
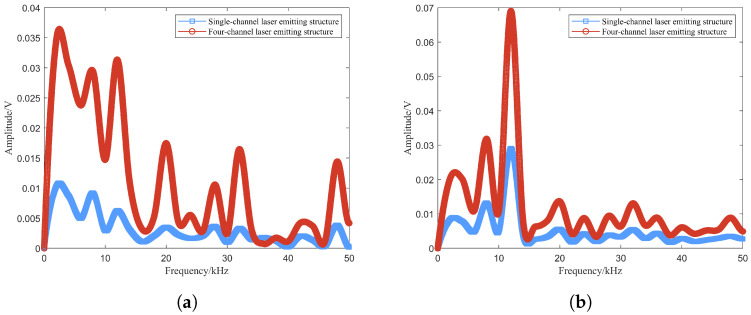
Spectrum and amplitude ratio of beat signals under the multiple laser structures: (**a**) Beat signal spectrum at the visibility of 12 m, (**b**) beat signal spectrum at the visibility of 15 m.

From Figure 16, the multiple-channel structure enhances the amplitude of the laser echo signal no more than the number of optical channels. Moreover, the effect of amplitude enhancement is affected by visibility, which specifically shows a decrease with the increase in visibility. Therefore, it is consistent with the actual situation.

## 5. Experiment

**Figure 17:**
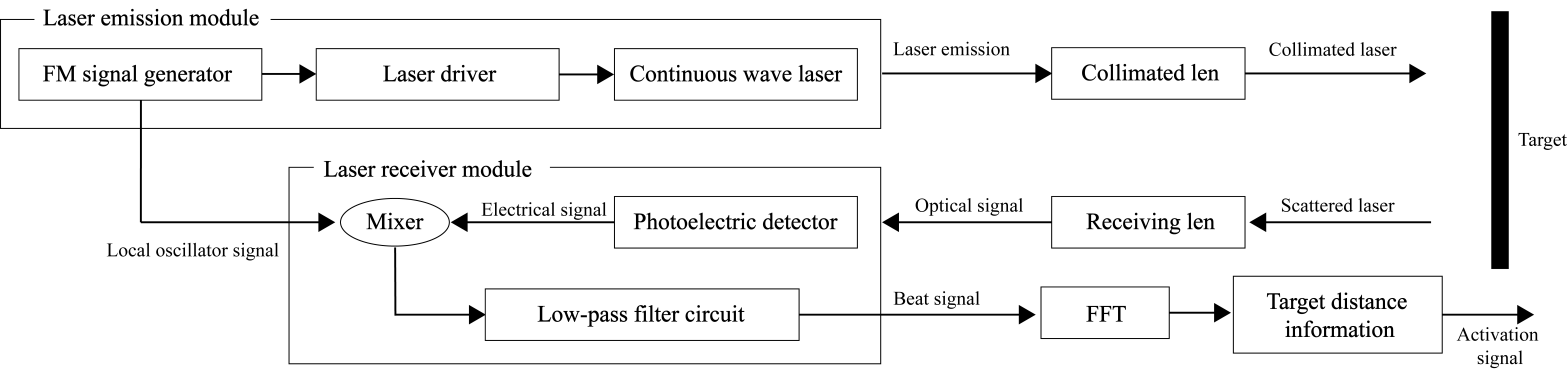
Principle of FMCW laser detection system operation.

**Figure 18:**
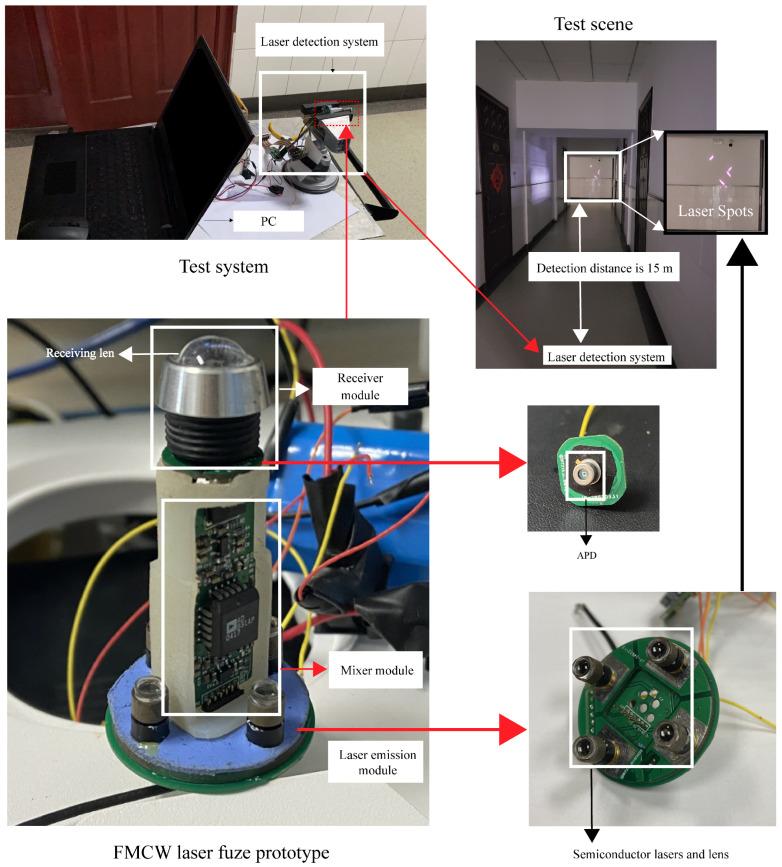
Laser detection system and laser spots.

The principle of FMCW laser detection system operation and four-channel laser emission structure of FMCW laser fuze prototype are shown in Figure 17 and Figure 18. The laser emission module includes an FM signal generation circuit, a laser driver circuit and a laser. The FM signal generation circuit is used to generate a triangular wave linear FM signal for the drive input of the laser drive circuit. A portion of this signal is directly input to the mixer as a local oscillator signal. The other part is used in the laser drive circuit to modulate the laser intensity and emit the laser outwardly. The received laser echo signal is converted photoelectrically by an avalanche photodiode (APD) and mixed with the local oscillator signal to output the beat signal by a low-pass filter [25]. For the four-channel laser emission structure, the four same continuous wave lasers are modulated by the laser intensity of four laser drive circuits. The four laser modulation signals of the transmitter circuit need to be controlled in phase synchronization to achieve coherent emission of multiple lasers.

**Figure 19:**
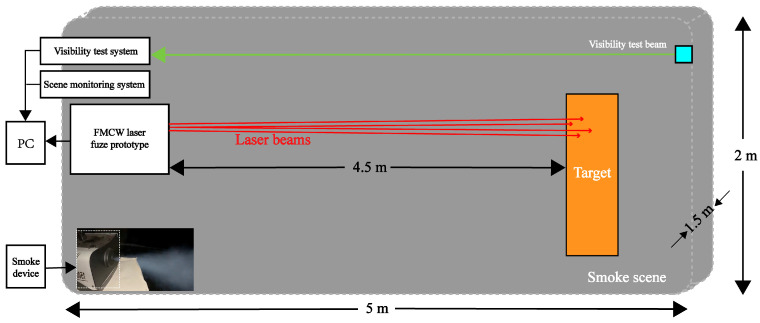
Schematic diagram of laser target characteristic test and set in the smoke.

In the laser fuze prototype, the semiconductor laser with a wavelength of 808 nm and a power of 500 mw is used at the laser transmitter, and an APD with a response wavelength of 400 nm to 1100 nm and reverse breakdown voltage of 120 V to 190 V is used at the laser receiver. The modulating signal is a triangular wave linear FM signal with a sweep bandwidth and a modulation period of 150 MHz and 0.5 ms, respectively. The lasers are uniformly and symmetrically distributed around the receivier with the same distance, and the delay time between the individual optical channels is set within 3 ns to control the distance deviation within 0.5 m based on Equation (16). The collimated laser beams are all controlled to be incident vertically toward the target surface and the divergence angle of the collimated lasers is not larger than 3 degrees. To ensure that multiple laser beams are incident on the target at equal distances, it is necessary to extract the target distances using the laser beat signals acquired by each laser in a smoke-free condition and compare them, respectively. In the case of large target distance differences, the laser collimation process needs to be repeated or the laser needs to be replaced until the requirements are met. Therefore, this section presents analysis in terms of the variation of the laser echo power under smoke conditions. A schematic diagram of laser target characteristic test and set in the smoke is shown in Figure 19, which mainly includes the FMCW laser detection system, the visibility test system, the smoke generation equipment and scene monitoring [26].

**Figure 20:**
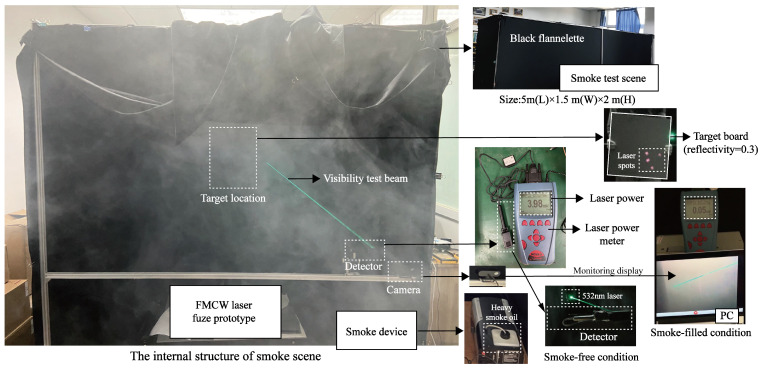
The actual smoke test scene.

**Figure 21:**
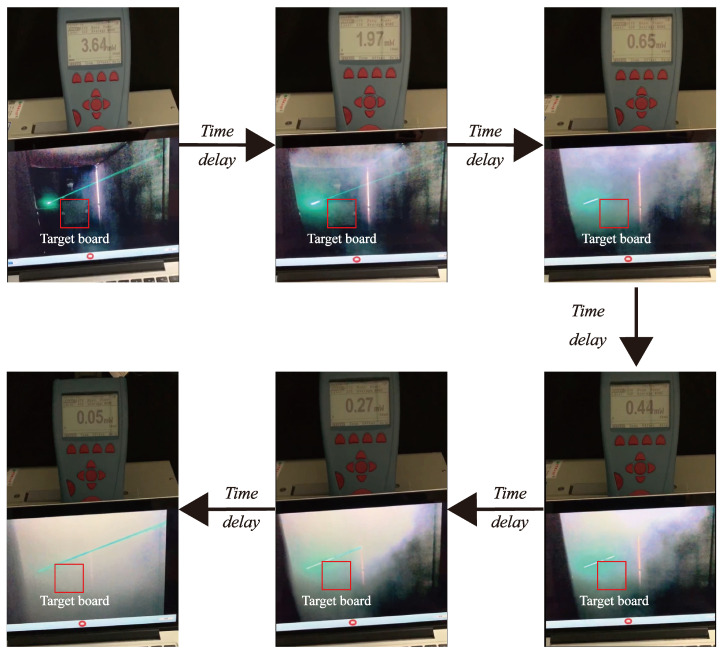
Generation of smoke environments and diffusion processes of particles.

The actual smoke test scene is shown in Figure 20. In the experiment, smoke is generated through the atomization of heavy smoke oil heated by an electronic smoke device, which is placed at a fixed edge location in an enclosed space. The smoke diffusion is completed through the process of continuous emission by the smoke device. By monitoring the smoke scene in Figure 21, the visibility test beam is observed to be clearest and continuous at optical powers of 0.05 mW, which reflect that the smoke particles are evenly distributed. On this basis, smoke scenes with different visibility levels can be obtained by time-lapse. Thus, it is possible to obtain the laser echo signal and the beat signal correspondingly with the laser fuze prototype.

The target board reflectivity is 0.3, and the distance between target and laser fuze prototype is set to 4.5 m. From Figure 20, it can be seen that the four-channel lasers are able to irradiate the target board. Based on Equation (16), the corresponding beat frequency can be calculated as

(23) $$f_{B}=\frac{4B}{cT_{m}}R=\frac{4\times 150\;\mathrm{MHz}}{3\times 10^{8}\;m/s\times 0.5\;\mathrm{ms}}\times 4.5\;m=18\;\mathrm{kHz}$$

The wavelength of the visibility test beam is set to 532 nm. According to the Beer–Lambert–Bouguer law [27], the visibility calculation formula can be expressed as

(24) $$V=L\frac{ln\epsilon }{ln\frac{I_{R}}{I_{0}}}$$

where ϵ is the predetermined threshold of laser transmission rate; its value can be set 0.05. *L* is the distance between laser transmitter and receiver; $I_{0}$ and $I_{R}$ are the emission and reception of laser power, respectively. $I_{0}$ and $I_{R}$ can be obtained from the laser power meter in the environment of smoke-free and smoke-filled conditions, respectively.

**Figure 22:**
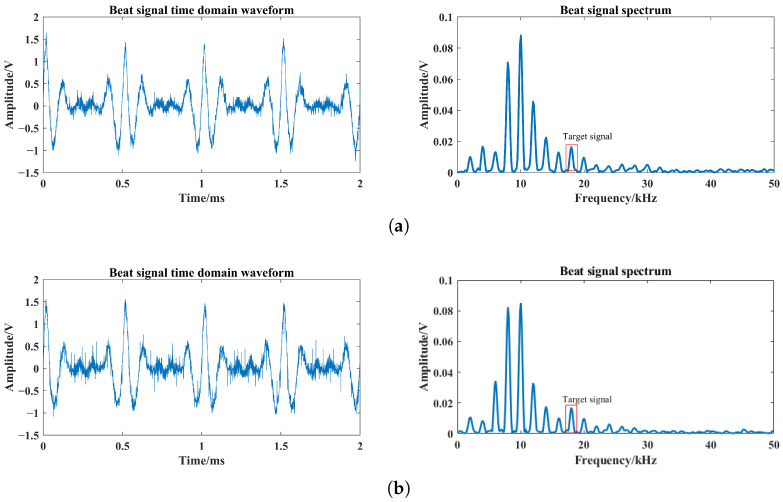
Beat signals and their spectrums based on single- and four-channel laser emission structures at visibility of 3.67 m: (**a**) Single-channel laser emission structure, (**b**) four-channel laser emission structure.

**Figure 23:**
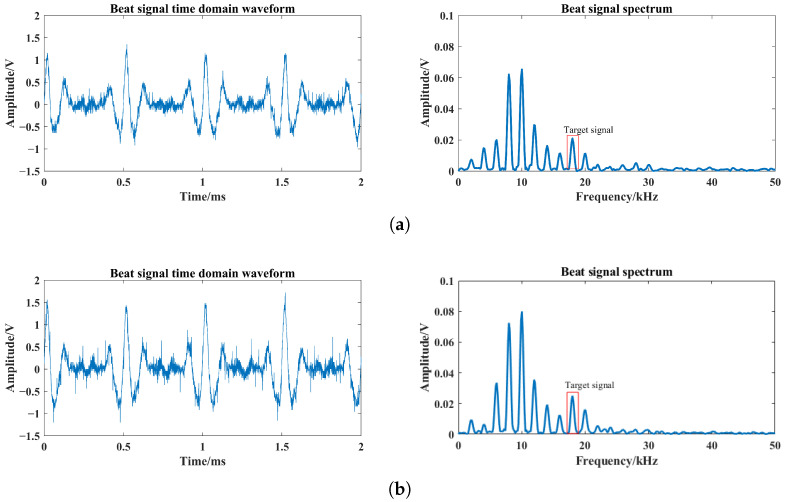
Beat signals and their spectrums based on single- and four-channel laser emission structures at visibility of 4.47 m: (**a**) Single-channel laser emission structure, (**b**) four-channel laser emission structure.

**Figure 24:**
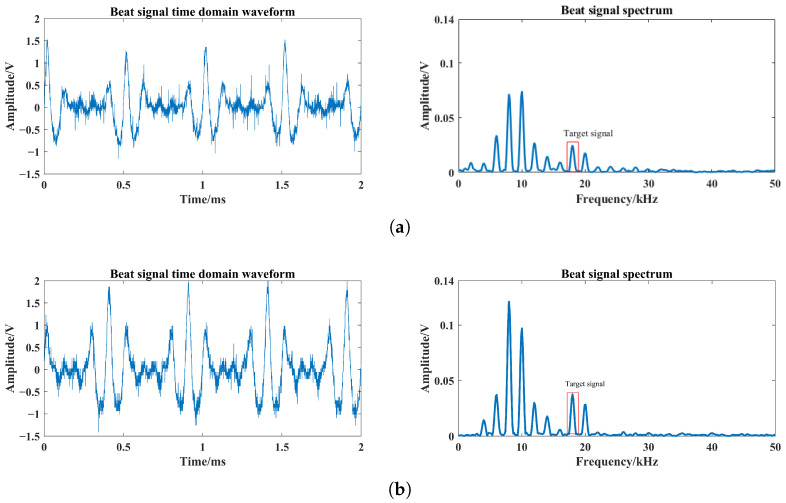
Beat signals and their spectrums based on single- and four-channel laser emission structures at visibility of 5.69 m: (**a**) Single-channel laser emission structure, (**b**) four-channel laser emission structure.

For single-channel and four-channel laser comparison test, the single laser uses one of the four lasers in the tests, which can directly reflect the enhancement of the target echo signal. Based on Figure 19, the laser echo power signal is obtained using the FMCW laser prototype under different optical power conditions. Among them, the beat signal spectrums at optical powers of 0.1 mW, 0.2 mW and 0.4 mW are shown in Figure 22, Figure 23 and Figure 24. The $I_{0}$ is set to 5 mW and the visibility corresponding to these optical powers is 3.67 m, 4.47 m and 5.69 m based on Equation (24), respectively.

From Figure 22, Figure 23 and Figure 24, smoke causes the beat signal spectrum to appear as interference signals at low-frequency locations. In the time domain, the target signal is completely submerged in the smoke interference signal; in the frequency domain, the target signal and the smoke interference signal are separated from each other.

According to the beat signal spectrum in Figure 22, Figure 23 and Figure 24, the peak values of the target echo signals are 0.016 V, 0.021 V and 0.025 V for the single-channel laser emission structure, and the peak values of the target echo signals are 0.017 V, 0.025 V and 0.038 V for the four-channel laser emission structure.

Therefore, as the number of optical channels increases, the peak value of target echo signal also increases, with an average growth rate of about 20%. Among them, the larger the visibility, the larger the increase in the peak value of the target echo signal, which means that the effect of optical channel number on the increase in laser echo power is positively correlated with smoke visibility. Moreover, at the visibility of 3.67 m, the peak value of the target echo signal also increases to a certain extent, which shows that it is feasible and effective to increase the optical channel number under very-low-visibility conditions.

**Figure 25:**
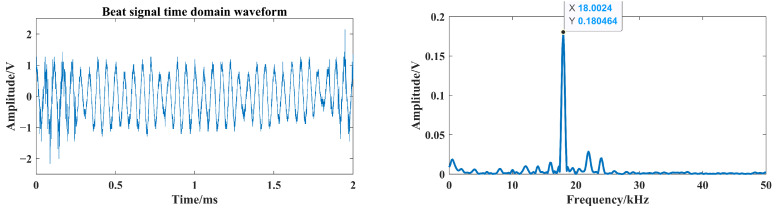
The beat signal and its spectrum in the smoke-free environment.

**Figure 26:**
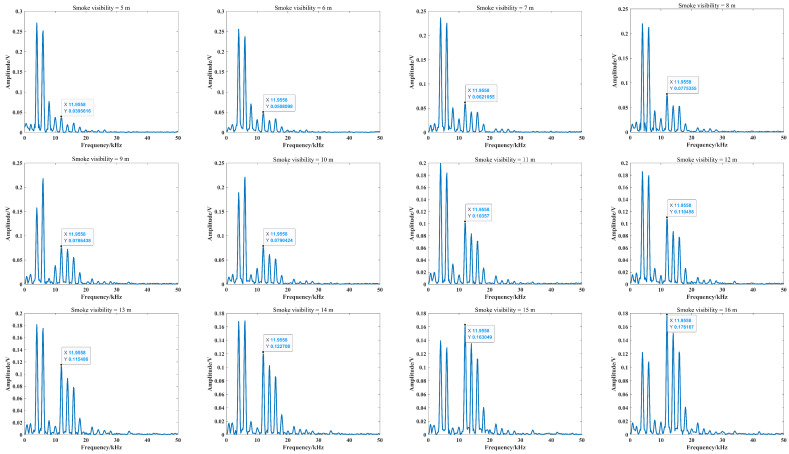
Beat signal spectrum at visibility from 5 m to 16 m in smoke scene.

In the smoke-free environment, the beat signal and its spectrum are shown in Figure 25, and it can be seen that the maximum peak of the spectrum is the same as the peak of the target echo signal and the beat frequency is about 18 kHz. According to Equation (23), the detection distance is consistent with the preset target distance, which demonstrates that the FMCW laser prototype can work properly. With the increased number of optical channels, the effect of laser echo power can be analyzed at a larger visibility range to represent the degree of stability of the FMCW laser prototype’s work in smoke. Moreover, the target distance is adjusted to 3 m to avoid the low amplitude of the target echo signal under low-visibility conditions. The spectrum of the laser beat signal at a visibility from 5 m to 16 m is shown in Figure 26.

**Figure 27:**
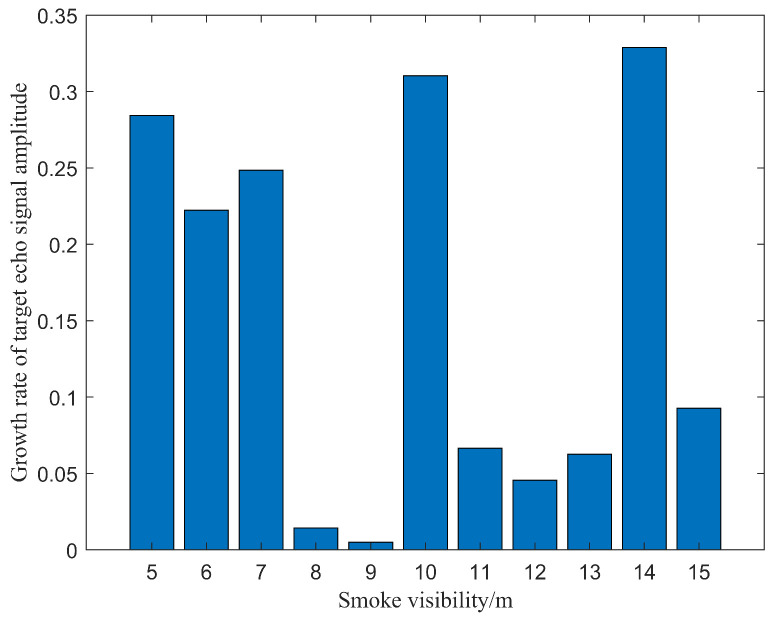
Growth rate of target echo signal amplitude at different visibilities.

Due to the uncertainty of spatial position distribution of smoke particles in the scene, the values of $n_{p1}$, $n_{p2}$ and $n_{p3}$ in Equation (13) also have uncertainties, which shows that multiple and mixed collisions between photons and smoke particles would be more likely to trigger scattering processes of photons. Thus, the growth rate of the target echo signal amplitude is also random, which is shown in Figure 27. However, this growth rate is always present, which reflects the continuous increase in the target echo signal amplitude and is consistent with objective changes. When the visibility is 15 m, the maximum peak value of the beat signal spectrum is the same as the peak value of the target echo signal, and the beat frequency is about 12 kHz. From Equation (21), the detection distance is also the same as preset target distance, which means that target distance can be calculated directly based on the frequency corresponding to the peak value of the spectrum at a visibility larger than 15 m.

## 6. Conclusions

In this paper, based on the scattering model of 3D particle collision, the fuze structure of multiple-channel laser emission is designed for the combined effect of particle features. Through studying the 3D collision process between photons and smoke particles, the FMCW laser multipath echo signal model based on 3D particle collision is established. Moreover, the simulation and analysis of the influence of different particle features on the laser echo signal are conducted by using the virtual particle simulation model based on Unity3D, and the results show that it is necessary to focus on the influence of the photon emission number and the smoke particle position on the laser echo signal and each component. For the case of joint particle features, simulation and similarity analysis under multiple-channel laser emission structures are performed. The results show that the average similarity increase in four- over single-channel emission structure is 102%. It shows that increasing the optical channels not only enhances the amplitude of the laser echo signal, but also effectively improves the anti-interference ability against the combined effect of multiple particle features.

Based on the FMCW laser prototype with a four-channel emission structure, the power variation of the laser echo signal under the smoke condition and the working properly in the smoke-free condition are experimentally verified. The results show that the increase in optical channels can indeed enhance the amplitude of the target echo signal, which is about 20% under the test conditions. Therefore, through the simulation and analysis of laser target characteristics under 3D particle collision, this can provide more reasonable and effective guidance to improve the detection performance of FMCW laser fuzes in the smoke, which can help the in-depth study of anti-smoke interference, target identification and structure optimization.

## Figures and Tables

**Table 1:** Main parameters of the virtual simulation.

Parameter	Value
Laser wavelength	0.808 μm
Sweep bandwidth	150 MHz
Modulation period	0.5 ms
Emission divergence angle	5 mrad
Receiving radius of lens	6 mm
Receiving field of view	45°
Target distance	3 m
Target reflectivity	0.3

## Data Availability

Data are all contained within the article and further inquiries can be directed to the corresponding author upon reasonable request.
